# Retraction: The protective role of MiR-206 in regulating cardiomyocytes apoptosis induced by ischemic injury by targeting PTP1B

**DOI:** 10.1042/BSR20191000_RET

**Published:** 2025-11-27

**Authors:** Yejun Yan, Hongweing Dang, Xin Xhang, Xia Wang, Xiaodong Liu

This article is being retracted from *Bioscience Reports* at the request of the Editor-in-Chief and the Editorial Board.

This follows the receipt of a notification from a reader, alerting the Editorial Board to concerns regarding the stylization and appearance of the Western blots. The authors were contacted with regard to the concerns and retraction but did not respond to the journal's queries, concerns raised, or the retraction notice.

Given the extent of the issues raised, the Editorial Board stands by the decision to retract the article.

